# Artemisinin-naphthoquine combination versus chloroquine-primaquine to treat vivax malaria: an open-label randomized and non-inferiority trial in Yunnan Province, China

**DOI:** 10.1186/1475-2875-12-409

**Published:** 2013-11-11

**Authors:** Hui Liu, Heng-lin Yang, Jian-Wei Xu, Jia-zhi Wang, Ren-hua Nie, Chun-fu Li

**Affiliations:** 1 Yunan Institute of Parasitic Diseases, Puer 665000, China; 2 Tengchong County Center for Disease Control and Prevention, Tengchong, China

**Keywords:** *Plasmodium vivax*, Artemisinin-naphthoquine, Efficacy, Safety

## Abstract

**Background:**

*Plasmodium vivax* is the main malaria parasite in China, and China is now making efforts to eliminate malaria by 2020. Radical cure of vivax malaria is one of challenges for malaria elimination. The purpose is to evaluate the efficacy and safety of artemisinin-naphthoquine (ANQ) versus chloroquine-primaquine (CQ-PQ) in treatment of vivax malaria in Yunnan Province, China.

**Methods:**

An open-label randomized and non-inferiority design, eligible patients with monoinfections of *P. vivax* were randomly assigned to receive either a total target dose of ANQ 24.5 mg/kg (naphthoquine 7 mg/kg and artemisinin 17.5 mg/kg), once a day for three days, or a total CQ dose of 24 mg base/kg, once a day for three days plus a PQ dose of 0.45 mg base/kg/day, once a day for eight days. Patients were followed up for one year. The difference in efficacy between ANQ and CQ-PQ was compared via Wilson’s test.

**Results:**

By day 42, the number of patients free of recurrence was 125 (98.4%; 95% Confidence interval, 94.4-99.8%) for ANQ arm and 123 (96.1%; 95%CI, 91.1-98.7%) for CQ-PQ, and non-significant (P = 0.4496). By day 365, the number was 101 (79.5%; 95%CI, 71.8-85.9%) for ANQ and 106 (82.8%; 95%CI, 75.1-88.9%) for CQ-PQ, and non-significant (P = 0.610). So the proportions of patients free of recurrence had no significant difference between ANQ and CQ-PQ groups by day 28, 42 and 365; compared with CQ-PQ, the side effect of ANQ was mild.

**Conclusion:**

ANQ is non-inferior to CQ-PQ in terms of patients free of recurrence, and safer than CQ-PQ.

## Background

*Plasmodium vivax* is the main malaria parasite in China. Yunnan Province is a vivax and falciparum malaria co-endemic region and one of two provinces with endemic falciparum malaria. China is now making efforts to eliminate malaria by 2020 [1,2]. Radical cure of vivax malaria is one of challenges for malaria elimination [3]. The treatment regimen, chloroquine (CQ) for blood stage infection and 8-aminoquinoline for liver stage parasite is often poorly adhered to [4,5]. Further, since CQ-resistant *P. vivax* was first described in 1989 in Papua New Guinea [6], the decline in the efficacy of CQ has been reported in some geographical sites [7].

In general, the use of artemisinin-based combination therapy (ACT) has been limited to patients with falciparum malaria and showed advantages in terms of adherence and safety [8-11]. However, with the high number of misdiagnoses in routine practice and the rise and spread of CQ-resistant *P. vivax*, there might be a compelling rationale for a unified ACT strategy for vivax and falciparum malaria in all co-endemic regions [12]. The use of ACT for patients with vivax malaria has been evaluated in China [13], Papua, Indonesia [14], Thailand [7] and Ethiopia [15]. These study results have documented that ACT was effective, safe and well-tolerated in the treatment of vivax malaria. In the context, an open-label randomized and non-inferiority trial was conducted to assess whether artemisinin-naphthoquine (ANQ) is as effective as chloroquine-primaquine (CQ-PQ), safer than CQ-PQ in treatment of patients with *P. vivax* monoinfections in Yunnan Province, China.

## Methods

### Patients

From February 2009 to December 2010, patients were recruited into our open-label randomized study at Tengchong County Center for Disease Control and Prevention, and Tengchong County Hospitals at the China-Myanmar border. The local transmission is from June to September in most parts of Tengchong County. Because of in malaria pre-elimination phase and an altitude higher than 2,000 m, there was only imported malaria and rarely local infection in recent years. Patients older than five years of age who weighed more than 15 kg and presented with single *P. vivax* malaria (parasite density 400–100,000 parasites per μL) were enrolled into the study. Patients were not admitted to the study if any the following criteria present: (1) pregnancy, (2) severe malaria, (3) having taken any anti-malarial drug within the past 14 days, (4) history of hypersensitivity to any of the study drugs, (5) severe dysfunction of kidney, liver and heart, (6) residence living at an altitude lower than 2,000 m, (7) unable to follow up.

### Allocation

As soon as confirmed patients were enrolled into the study, they were randomly assigned to receive either ANQ or CQ-PQ regimens. A researcher, who did not have a role in recruitment, put sealed envelopes in blocks of 50 (25 ANQ and CQ-PQ respectively) in a box, and an enrolled patient drew an envelope from it to achieve treatment allocations in equal numbers. When the box was empty, another 50 envelopes were added.

### Drugs

ANQ was registered by Food and Drug Administration, China as GYZZ H20050270 and licensed to Kunming Pharmaceutical Corp to produce. A tablet of ANQ is composed of 50 mg naphthoquine and 125 mg artemisinin. CQ was registered as GYZZ H31020423 and PQ as GYZZ H2005984 in China. CQ-PQ was produced and pre-packed by Shanghai Sino-West Pharmaceutical Corp. Both ANQ and CQ-PQ were administered following the anti-malarial drug policy of China. Based on the hypothesis that a three day dosing could reduce recurrence of *Plasmodium vivax,* ANQ was given once a day for three days, with a total target dose of 24.5 mg/kg (naphthoquine 7 mg/kg and artemisinin 17.5 mg/kg). CQ was given once a day for three days with a total target dose of 24 mg base/kg and PQ was offered once a day for eight days with a dose of 0.45 mg base/kg/day.

### Follow-up

The researchers visited all patients every 8 hrs for the first three days. Axillary temperatures were measured every 8 hrs after treatment until after 48 hrs of fever clearance. Thick and thin blood smears were taken and examined every 8 hrs in each active visit, and then day 7, 14, 21 and 28 respectively. The subsequent PQ doses of CQ-PQ groups were given under supervision of patient caretakers one hour after supper and before going to bed from the fourth day. Patients received the remaining PQ doses packed by aluminium-plastic foil and were instructed clearly about their subsequent treatment, emphasizing the importance of taking drugs after food and before going to bed, and taking their medicines even when their symptoms had subsided. The patients were asked to return for treatment in any case that they had dark urine; this was done instead of testing for G6PD deficiency because of shortage of reagent kit and equipment supplies. Patients were visited and interviewed every month; meanwhile blood smears were done during the monthly visits, and asked to return for screening and treatment at any time, if they became ill. If a patient had fever, blood smears were prepared and examined for malaria at any time. Microscopists examining blood films were unaware of treatment allocation. If any patients were positive of *P. vivax* again, they were retreated with CQ (24 mg base/kg) for three days and PQ (0.25 mg base/kg/d) for 14 days. Patients were observed and interviewed with semi-structured questionnaires in depth for adverse reactions and compliance during administering each dose and follow-up visits on days 1, 2, 3, 7, 14, 21 and 28. Patients were followed up for one year through monthly visits.

### Laboratory methods

Malaria blood films were stained with Giemsa, and both asexual parasites and gametocytaemia were counted per 500 white blood cells. The number of parasites was calculated as per μl of blood by the level of 8,000 of leukocyte per μl [16]. Parasite clearance was defined as no any parasite per 500 white blood cells by two continuous every 8-hr microscopy and gametocyte clearance defined by the same method. Fever clearance was defined as axillary temperatures < 37.1°C in duration of 24 hrs. Cure was defined as elimination of the symptoms and asexual blood stages of the malaria parasites that caused the patient to seek treatment. Recurrence was defined as reappearance of asexual parasitaemia following treatment caused by a recrudescence, a relapse or a new infection [4].

### Study end points

The primary end point was the proportion of patients free of recurrence at day 42, and the secondary end points included at day 28 and 365, the parasite and fever clearance times, and the adverse events reported by patients during follow-up.

### Statistical analysis

Based on calculation recommended by literature [17], a sample size of 120 patients per study group allowed a cure rate of 90% per group to be estimated with 5% precision, and allowed estimation of effectiveness equivalence with a maximum allowable difference of 10% (90% power and 95% confidence) between groups with a follow-up drop-out rate of up to 10%.

The difference of patients free of recurrence and its two-sided 95% confidence intervals between ANQ and CQ-PQ arms were calculated via Wilson’s test. Proportions were compared by using Yates corrected *x*^2^ tests. Mean fever and parasite clearance time were compared by covariance.

### Ethical approval

The study was reviewed and approved by The Academic Board of Yunnan Institute of Parasitic Diseases (YIPD) in China as protocol 200807. Approval was also obtained from YIPD’s ethics committees. The purpose of the study was explained and approval was sought from patients and their caretakers. Informed written consent was obtained from patient or caretakers of child patients. All results were kept confidential and were unlinked to any identifying information.

## Results

**Figure 1:**
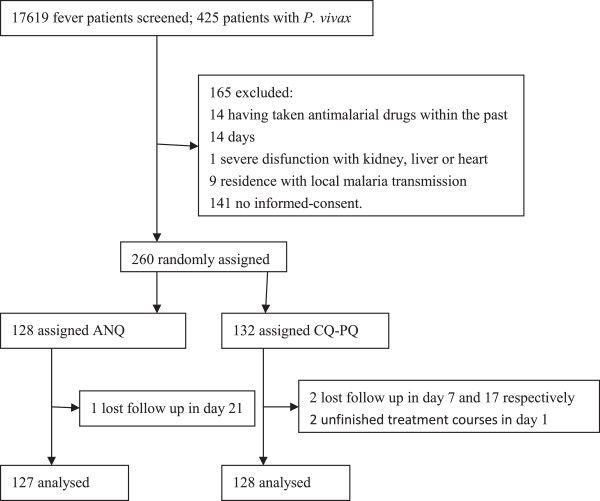
Trial profile.

The patient proportion of rejecting enrolment was high (35.2%). When these patients knew that they might take CQ-PQ for eight days, they were not willing to be involved in the trial. Of 17,619 fever patients screened for malaria, 425 had *P. vivax*. 401 met the enrollment criteria, but 141 did not agree to participate in the study. 260 were randomly assigned to one of treatment groups. 128 (49.2%) and 132 (50.8%) received ANQ and CQ-PQ respectively. Three (1.2%) have not completed the 365 day follow-up and two (0.8%) withdrew from the study on day 1 (Figure 1). Baseline characteristics were similar between the two treatment groups. 228 (89%) of all patients enrolled were male and 232 (91%) were older than 16 years (Table 1). All patients were Chinese citizens. Most of them went a neighbouring country and got infected there, and then came back China for treatment. According to exclusion criteria (6), patients who went go back and forth Myanmar during the study period were not admitted to the study because of being unable to follow up. So most patients stayed in their homeland during the study period, three patients who are lost follow up might go back Myanmar.

**Table 1:** Baseline characteristics of patients in Yunnan Province, China

	**ANQ (n = 127)**	**CQ-PQ (n = 128)**	**All (n = 255)**
**Sex**			
Male	118 (93%)	110 (86%)	228 (89%)
Female	9 (7%)	18 (14%)	27 (11%)
**Age groups (years)**			
5-16	2 (2%)	20 (16%)	22 (9%)
>16	125 (98%)	107 (84%)	232 (91%)
**Mean (SD) weight, kg**	**60.7(9.9)**	**52.6 (17.7)**	**56.6 (14.9)**
**Mean (SD) height, cm**	**167.6 (4.9)**	**159.8 (18.7)**	**163.6 (14.2)**
**Geometric mean parasite count**	**3899**	**2546**	**3147**
**Range (per μl)**	424-47100	400-33371	400-47100
**Gametocytaemia on admission**	**127 (100%)**	**128 (100%)**	**255 (100%)**
**Duration of illness before presentation (hr)**			
Mean (± SD)	51.8 (15.6)	52.2 (13.7)	52.0 (14.5)
Range	17-113	21-103	17-113
**Body temperature (°C)**			
Mean (± SD)	38.4 (1.3)	38.2 (1.2)	38.3 (1.2)
Range	36.7-41.0	36.6-40.7	36.6-41.0
**Haemoglobin values (g/l)**			
Mean (± SD)	140.3 (19.1)	145.5 (17.3)	142.7 (19.2)
Range	96-168	102-181	96-181
**Pulse rates (/min)**			
Mean (± SD)	76.3 (7.3)	76.7 (6.5)	75.5 (6.4)
Range	71-91	62-90	62-91

The proportion of patients with recurrence in the ANQ group were not significant (P = 0.4496) to that in the CQ-PQ arm by day 42 (Table 2). Neither group had any recurrence by day 28. The ANQ group had two recurrences by day 42, respectively on day 36 and 38; the CQ-PQ group had five recurrences, one respectively on day 30, 35 and 41, and two on day 31. The ANQ group had 26 (20.5%) recurrences by day 365, one patient had two recurrences with a 69-day interval, and 25 had only one. The CQ-PQ group had 22 (17.2%) recurrences, two patients had two recurrences and 20 had only one; the intervals of two recurrences for the two patients were respectively 153 and 121 days. Most of recurrences occurred between day 43 and 98, the median time of ANQ to recurrence was 77 days (range, 29–347 days) and CQ-PQ arm 85 days (range, 24–357 days) (Table 3).

**Table 2:** Therapeutic responses of patients in Yunnan Province, China

	**ANQ (n = 127)**	**CQ-PQ (n = 128)**	**P-value**	**Difference**
**Fever clearance times (hr)**				
Mean (± SD)	26.9 (8.2)	39.8 (11.4)	<0.0001	
Range	12-72	17-64		
**No. with asexual parasitaemia on day 1 (24 hr)**	25 (19.7%; 95%CI, 13.2-27.7%)	76 (59.4%; 95%CI, 50.3-68.0%)	<0.0001	0.397 (95%CI, 0.281-0.498)
**No. with asexual parasitaemia on day 2 (48 hr)**	25 (19.7%; 95%CI, 13.2-27.7%)	58 (45.3%; 95%CI, 36.5-54.3%)	<0.0001	0.256 (95%CI, 0.142-0.361)
**50% parasite clearance times (hr)**				
Mean (± SD)	8.3 (2.4)	12.8 (6.9)	0.023	
Range	7-17	8-32		
**Parasite clearance times (hr)**				
Mean (± SD)	26.0 (5.6)	37.4 (11.9)	<0.0001	
Range	15-48	15-64		
**Gametocyte clearance times**				
Mean (± SD)	26.7 (7.5)	36.2 (12.5)	<0.0001	
Range	8-48	8-64		
**Proportion of patients free of recurrence by day 28**	127 (100%; 95%CI, 97.1-100%)	128 (100%; 95%CI, 97.2-100%)	-	0 (95%CI, -0.029-0.029)
**Proportion of patients free of recurrence by day 42**	125 (98.4%; 95%CI, 94.4-99.8%)	123 (96.1%; 95%CI, 91.1-98.7%)	0.4496	0.0233 (95%CI, -0.022-0.074)
**Proportion of patients free of recurrence by day 365**	101 (79.5%; 95%CI,71.8-85.9%)	106 (82.8%; 95%CI, 75.1-88.9%)	0.610	0.0328 (95%CI, -0.064-0.129)

**Table 3:** Recurrence of patients in 365 day in Yunnan Province, China

	**ANQ (n = 127)**	**CQ-PQ (n = 128)**	**P-value**
**Patients with parasite reappearance in one year**	**26 (20.5%; 95%CI, 14.1-28.2%)**	**22 (17.7%; 95%CI, 11.1-24.9%)**	**0.61**
Day 0-28	0 (0.0%)	1 (0.8%)	-
Day 29-42	2 (1.6%)	5 (3.9%)	0.45
Day 43-98	20 (17.7%)	13 (10.2%)	0.18
Day 99-175	2 (1.6%)	1 (0.8%)	0.95
Day 176-245	0 (0.0%)	1 (0.8%)	-
Day 246-365	2 (1.6%)	1 (0.8%)	0.95

All 255 patients cleared parasitaemia by day 3, ANQ treatment group by hour 48 and CQ-PQ by hour 64. 50% parasite, full parasite, gametocyte clearance times of ANQ were shorter than of CQ-PQ. ANQ cleared parasitaemia very rapid, the proportion of patients with parasitaemia at 24 hrs after therapy was significantly lower than of CQ-PQ. The fever clearance time (FCT) of ANQ was significantly shorter than of CQ-PQ group (Table 2). 49 (19.2%) patients reported adverse events during the study (Table 4). 9 (7.1%) in ANQ arm and 7 (5.5%) in CQ-PQ group had nausea and anorexia within the first hour respectively. However, whether the drugs caused the side effect could not be confirmed. The proportion with adverse events were similar (RR 1.05; 95%CI, 0.63-1.74; P = 0.97) between the two groups, but side effect of the ANQ was mild. In CQ-PQ group, two patients withdrew because of the serious adverse effect. They were both male, 33 and 39 years of age. Their body temperatures were respectively 37.3°C and 37.0°C (not at time of malaria attack) when they presented, however, the temperature of patient 1 climbed to 39°C after four hrs taking CQ-PQ and patient 2 to 38.6°C after four and half hrs. Their baseline hemoglobin was respectively 149 g/l and 136 g/l. After the two patients took the first dose of PQ (22.5 mg/person), they felt more uncomfortable than prior to taking CQ-PQ for malaria treatment and their urines were the color of black tea. The result of haemoglobinuria test were respectively “++” (≥ 2 g/l) for patient 1 and “+++” (≥3 g/l) for patient 2. Following the guideline on treatment of G6PD deficiency, they stopped use of PQ. They were treated with ANQ and excluded them from the study. Their urines became normal without special treatment after 24 hrs not taking PQ. The two patients were Jingpo and Dai ethnical minority respectively. Their personal or family history of haemolysis/haemoglobinuria could not be investigated because they could not provide related information. Based on the above, the two patients were supposed to be G6PD deficiency and the primaquine caused their haemoglobinuria.

**Table 4:** Number of patients reporting side-effects at any time point after drug administration in Yunnan Province, China

	**ANQ (n = 127)**	**CQ-PQ (n = 128)**	**P-value**
**Adverse response**	**25 (19.7%)**	**24 (18.8%)**	**0.97**
Dizziness	1 (0.8%)	1 (0.8%)	-
Nausea	9 (7.1%)	7 (5.5%)	0.59
Anorexia	9 (7.1%)	7 (5.5%)	0.59
Diarrhoea	2 (1.6%)	1 (0.8%)	0.99
Abdominal pain	1 (0.8%)	1 (0.8%)	-
Palpitations	0 (0.0%)	1 (0.8%)	-
Headache	0 (0.0%)	2 (1.6%)	-
Vomiting	3 (2.4%)	2 (1.6%)	0.99
Haemolysis	0 (0.0%)	2 (1.6%)	-

## Discussion

The study results showed that ANQ had similar efficacy to CQ-PQ in terms of patients free of recurrence, and better tolerated and safer than CQ-PQ. Naphthoquine has a half-life of 40.93 hrs. The cure rate of naphthoquine phosphate (NP, 100%) was higher than CQ (74.3%) by day 42, but longer fever clearance times (FCT) and parasite clearance times (PCT) than of CQ [18]. ANQ was evaluated to overcome the shortage of NP in FCT and PCT in Hainan Province of China from May 1999 to October 2000. The cure rate of ANQ was 100% by day 42, and FCT and PCT was shorter than of CQ [19]. In Papua New Guinea, ANQ has been used for *P. vivax* infections now. The study showed that the lower single ANQ dose was associated with relatively frequent recurrence of parasitaemia [20]. In Thailand, dihydroartemisinin-piperaquine (DP) was evaluated in the treatment of vivax malaria. The results showed that the cumulative risk of recurrence with *P. vivax* was significant lower in DP recipients than in CQ [7]. In southern Papua, Indonesia, a study results showed that recurrence of vivax malaria occurred in 38% of patients given artemether-lumefantrine and 10% given DP [14]. All the studies showed that ACT cleared *P. vivax* very rapidly and had high cure rates. This coincides with the viewpoint that *P. vivax* is more sensitive than *P. falciparum* to the artemisinin derivatives [4].

Artemisinin has a short half-life. A single dose or a two-day dosing of ANQ is usually for treatment of *Plasmodium falciparum*. The finding in Papua New Guinea showed that the lower single ANQ dose was associated with relatively frequent recurrence of *P. vivax*[20]. Considering these factors and that patients can easily adhere to three-day regimens, dosing ANQ for three days was chosen in the study.

The efficacy of CQ in the treatment of *P. vivax* infections was declining on the Thai-Myanmar border [7] and in Vietnam [21]. The cumulative risk of recurrence with *P. vivax* at nine weeks was 79.1% in patients treated with only CQ and 54.9% treated with dihydroartemisinin- piperaquine on the Thai-Myanmar border [7]. As a primary result of the study, the proportions of patients free of recurrence between ANQ and CQ-PQ groups had no difference even by day 365. The cumulative recurrence rates with *P. vivax* at 365 days were respectively 21.05% in patients treated with ANQ and 17.2% treated with CQ-PQ. This indicated that PQ and NP significantly reduced recurrence with *P. vivax.* This might attribute to two reasons. One is that NP has a long half-life despite ANQ cannot kill the liver stages, whereas CQ-PQ can; the other is that no evidence documented that less than 14 days PQ can radically cure *P. vivax*[4]. Despite the total dose of PQ with the 0.45 × 8 day (=3.6 mg) regimen is similar to the 0.25 mg/kg qd × 14 days (=3.5 mg) regimen recommended by WHO, the longer time of treatment seems to be important for killing hypnozoites. As a limitation, the subsequent PQ doses from day 4 to 8 were given under supervision of patient caretakers, not under supervision of researchers; despite the patients or/and their caretakers said the patients completed taking PQ regimen, the researcher could ensure that they really took PQ completely. It is possible that the people with relapse took a smaller dose of total PQ. As one of exclusion criteria for both ANQ and CQ-PQ groups, patients who went go back and forth Myanmar during the study period were not admitted to the study because of being unable to follow up, so it is not necessary to keep on at the effect of re-infection on the study result.

ANQ had a mild side effect. However, two patients could not complete the treatment because of PQ toxicity. 2064 persons were screened for G6PD deficiency in China-Myanmar border region in July 2009. The result was 2.3% (95%CI, 1.7-3.0%) of prevalence of G6PD deficiency (not published). These showed that using CQ-PQ has an intrinsic problem in the region. Patients of vivax malaria can obtain 8-day CQ-PQ regimen free from public health sector in China. As a reality, malaria patients want to get rid of symptoms as quick as possible. They commonly seek artemether or pyronaridine injection by self-medication or from private clinics because they considered injection working better and faster than oral tablets, and intolerability of the 8-day CQ-PQ regimen. All the update studies showed that ACT was well tolerated with no serious adverse events [7-14,20]. There is rarely local malaria transmission in the study area due to low temperature attributed to high altitude, malaria patients are from migrants, most of them are adult male, so the issue of re-infection was omitted during study period.

## Conclusion

In terms of efficacy, the three-day ANQ regimen is as effective as the 8-day CQ-PQ, safer and more acceptable than CQ-PQ, but ANQ does not prevent relapse completely. ANQ is a great blood schizonticide for *P. vivax* infections and is a good option for Yunnan Province of China if people do not want to take PQ.

## Competing interests

The authors declare that they have no competing interests.

## Authors’ contributions

HL, H-L Y designed the study and developed the protocol. J-W X and HL analyzed and interpreted the data. HL supervised the clinical trial. HL, J-Z W, R-H N and C-F L conducted the clinical trial, and entered the data. J-WX and HL wrote the first draft of the paper. All authors read and approved the final manuscript.
